# Complete genomes of *E. coli* with diverse K antigens

**DOI:** 10.1128/mra.00013-26

**Published:** 2026-05-29

**Authors:** Carine Roese Mores, Christopher M. Field, Hans-Joachim Ruscheweyh, Emma Slack, Timothy G. Keys, Shinichi Sunagawa

**Affiliations:** 1 Department of Biology, Institute of Microbiology and Swiss Institute of Bioinformatics, ETH Zurich 31154 , Zurich, Switzerland; 2 Department of Health Sciences and Technology, Institute of Food, Nutrition and Health, Institute of Food, Nutrition and Health, ETH Zurich 27219, Zurich, Switzerland; University of Maryland Baltimore, Baltimore, Maryland, USA

**Keywords:** *Escherichia coli*, surface antigens, capsular polysaccharide

## Abstract

*Escherichia coli* is a diverse species with commensal and pathogenic lifestyles. Capsular polysaccharides (K antigens) are prominent surface structures, important virulence factors, and targets for immune recognition. The complete genomes of 68 strains with serologically distinct capsule types were sequenced using a combination of PacBio/Oxford Nanopore and Illumina technologies.

## ANNOUNCEMENT

The capsular polysaccharide (CPS, or K antigen) is an important virulence factor of pathogenic *Escherichia coli* strains (1, 2). However, our understanding of CPS epidemiology remains limited by the cost- and labor-intensive nature of *in vitro* serotyping (3). While laboratories increasingly adopt whole genome sequencing for *in silico* phenotyping, inferring *E. coli* capsule types are currently hindered by a lack of data linking serotypes to genomes. Here, we sequenced the genomes of 68 *E. coli* strains obtained from reference culture collections (Statens Serum Institut, Denmark, and Culture Collection, University of Gothenburg, Sweden).

Cryostocked strains were streaked on LB agar plates and incubated at 37°C for 24 h. Single colonies were transferred to LB and incubated at 37°C for 24 h, and genomic DNA was extracted from cell pellets using NucleoBond AXG 20 columns with the Buffer Set III (Macherey-Nagel, Düren, Germany). Concentration, purity, and fragment size were measured using a Qubit fluorometer, a Nanodrop spectrophotometer, and a Femto Pulse system, respectively (Thermo Fisher Scientific, USA, Agilent Technologies, USA). For Illumina sequencing, libraries were generated using unsheared DNA with the Nextera XT Kit and sequenced (2 × 150 bp) on the NovaSeq 6000 system. Raw reads were quality-controlled to remove adapter (ktrim = r k = 23 mink = 11 hdist = 1) and PhiX sequences (k = 31 hdist = 1) and to trim low-quality bases using the bbduk.sh command of BBmap v38.87 (4) with parameters: trimq = 14, qtrim = r, maq = 20, maxns = 0, and minlength = 45. For PacBio sequencing, DNA was sheared to an average size of 7–10 kb using a Megaruptor 3 device (Diagenode) and was not size-selected. Libraries were constructed using the SMRTbell Prep Kit 3.0 and sequenced on the Sequel IIe system using the Sequencing Kit 2.0 (one SMRT Cell 8 M, HiFi mode, 15 h). QC was performed during the run using Control SW Version 11.0.0.144,466. Oxford Nanopore Technology (ONT) libraries were prepared using unsheared, non-size-selected DNA with the rapid barcoding kit (SQK-RBK114) and sequenced using a MinION FLO-MIN114 flow cell (MinION Mk1C device, 72 h). Guppy v6.4.6 (5) was used for basecalling, demultiplexing, and quality control of the ONT reads. Assemblies were performed with Flye v2.9 (6) with the “pacbio-hifi” and “nano-raw” options for PacBio (“Q20+”) and ONT (“pass”) reads, respectively. Quality-controlled Illumina reads were used to polish all assemblies using Pilon v1.23 (7). All tools were run with default parameters unless otherwise specified.

The genomes exhibited a size range from 4.5 to 5.2 Mb (Table 1) and were annotated with NCBI PGAP v2024-07-18.build7555 (8) with default settings. Visualization of assembly graphs and verification of completeness were performed using Bandage v0.9.0 (9). Overall, the reported genomes provide unprecedented access to study the molecular basis underlying the diversity of K antigens in *E. coli*.

**TABLE 1:** Genome assembly statistics and accession numbers

Capsular serotype	Strain identifier^*a*^	No. of short reads	Long-read technology	No. of long reads	Long-read N50 (bp)	Genome length (bp)	GC content (%)	Genome coverage	ECEs	GenBank accession no.	Short-read accession no.	Long-read accession no.
K1	SSI 85361	8,843,528	PacBio	37,200	9,485	4,961,657	50.71	61	1	GCA_977947085	ERR14050185	ERR14041770
K2a	CCUG 27	11,751,454	ONT	57,349	25,061	5,125,785	50.54	49	1	GCA_977947715	ERR14050194	ERR14041773
K2ab	SSI 85362	9,401,764	PacBio	38,443	9,226	4,700,954	50.53	68	1	GCA_977947245	ERR14050196	ERR14041788
K3	SSI 85363	11,370,150	PacBio	34,889	9,383	514,176	50.41	57	2	GCA_977947575	ERR14050206	ERR14041791
K4	SSI 85364	14,322,598	PacBio	36,172	9,928	4,763,144	50.71	66	1	GCA_977947105	ERR14050210	ERR14041794
K5	SSI 85365	10,278,192	ONT	88,384	17,421	5,227,725	50.75	41	3	GCA_977947595	ERR14050213	ERR14041797
K6	SSI 81964	8,440,706	ONT	30,422	21,162	5,167,108	50.42	31	1	GCA_977947035	ERR14050216	ERR14041799
K7	SSI 81965	11,578,402	PacBio	41,401	9,538	4,736,535	50.63	76	1	GCA_977947585	ERR14050218	ERR14041800
K8	SSI 85366	10,442,820	PacBio	45,287	9,376	4,750,763	50.92	81	0	GCA_977947505	ERR14050223	ERR14041802
K9	SSI 85367	7,893,366	ONT	92,184	20,616	4,913,262	50.78	50	0	GCA_977946355	ERR14050225	ERR14041805
K10	SSI 85368	9,330,748	PacBio	30,281	9,653	4,883,361	50.79	53	2	GCA_977947665	ERR14050229	ERR14041806
K11	SSI 85369	9,423,246	ONT	88,383	19,801	4,573,209	50.74	53	4	GCA_977947875	ERR14050233	ERR14041809
K12	SSI 81966	3,639,474	ONT	96,876	15,401	5,286,215	50.45	40	2	GCA_977946375	ERR14050238	ERR14041813
K13	SSI 81967	11,463,720	PacBio	26,200	9,413	5,078,894	50.46	42	0	GCA_977947965	ERR14050242	ERR14041814
K14	SSI 85370	9,456,546	PacBio	35,501	9,568	4,581,132	50.71	68	0	GCA_977946545	ERR14050246	ERR14041815
K16	SSI 85371	8,768,754	PacBio	37,824	9,719	5,007,734	50.49	62	2	GCA_977947945	ERR14050249	ERR14041818
K17	SSI 85372	8,458,344	PacBio	37,922	9,152	4,600,220	50.8	69	1	GCA_977947915	ERR14050252	ERR14041819
K18a	SSI 81968	8,888,756	PacBio	35,780	9,325	5,068,856	50.71	57	2	GCA_977946865	ERR14050254	ERR14041821
K18ab	SSI 85373	486,230	ONT	27,461	15,050	5,069,166	50.72	35	2	GCA_977946875	ERR14050255	ERR14041824
K19	SSI 85374	8,343,316	PacBio	41,642	9,692	4,880,777	50.7	75	0	GCA_977947905	ERR14050258	ERR14041828
K20	SSI 85375	10,728,454	PacBio	38,318	9,860	4,991,724	50.86	70	0	GCA_977947155	ERR14050261	ERR14041829
K22	CCUG 11329	7,852,506	PacBio	38,024	8,664	5,085,183	50.72	56	2	GCA_977947955	ERR14050263	ERR14041831
K23	SSI 81970	8,639,116	ONT	95,956	21,074	5,125,131	50.42	92	0	GCA_977946625	ERR14050266	ERR14041833
K24	SSI 81971	11,535,816	ONT	58,864	34,261	5,105,026	50.46	59	0	GCA_977946345	ERR14050269	ERR14041836
K26	SSI 81972	9,819,104	PacBio	36,939	10,507	4,555,035	50.91	73	1	GCA_977946425	ERR14050272	ERR14041838
K27	SSI 81973	10,544,150	PacBio	31,751	11,071	4,646,608	50.93	68	0	GCA_977946285	ERR14050275	ERR14041842
K28	SSI 81974	7,338,250	PacBio	31,365	10,747	4,654,761	51.05	65	0	GCA_977946325	ERR14050278	ERR14041843
K29	SSI 81975	3,251,212	ONT	23,279	37,588	4,828,683	50.79	39	2	GCA_977946365	ERR14050279	ERR14041845
K30	SSI 81976	9,417,840	PacBio	37,295	9,715	4,808,187	50.87	64	2	GCA_977946635	ERR14050282	ERR14041847
K31	SSI 81977	10,874,260	PacBio	32,419	11,105	4,521,692	51.04	71	1	GCA_977947365	ERR14050286	ERR14041849
K32	SSI 81978	7,818,130	PacBio	31,234	10,974	4,943,816	50.84	62	1	GCA_977946405	ERR14050288	ERR14041851
K33	SSI 81979	8,403,202	PacBio	30,205	11,803	4,597,013	50.98	67	1	GCA_977946605	ERR14050291	ERR14041853
K34	SSI 81980	5,957,044	PacBio	29,718	11,398	4,529,990	50.91	67	0	GCA_977946575	ERR14050294	ERR14041857
K35	SSI 81981	6,432,850	PacBio	30,641	9,039	4,610,049	50.91	52	1	GCA_977946905	ERR14050296	ERR14041858
K36	SSI 81982	6,876,172	PacBio	42,137	9,185	4,897,268	50.83	72	0	GCA_977946735	ERR14050324	ERR14041861
K37	SSI 81983	8,387,472	PacBio	39,222	8,648	4,597,789	50.97	68	0	GCA_977946935	ERR14050363	ERR14041863
K38	SSI 81984	9,360,838	PacBio	29,217	8,653	4,842,863	51.08	47	0	GCA_977946685	ERR14050365	ERR14041864
K39	SSI 81985	10,294,304	PacBio	37,282	9,274	4,682,936	50.84	67	1	GCA_977946655	ERR14050368	ERR14041865
K40	SSI 81986	10,043,060	PacBio	33,985	9,822	4,542,529	50.81	66	1	GCA_977946775	ERR14051037	ERR14041867
K41	SSI 81987	8,383,774	PacBio	37,163	9,549	4,797,978	50.89	67	1	GCA_977946705	ERR14051039	ERR14041869
K42	SSI 81988	7,951,674	PacBio	37,978	9,526	4,791,211	50.89	67	1	GCA_977946795	ERR14051042	ERR14041870
K43	SSI 81989	4,254,874	PacBio	37,266	9,959	4,827,059	50.83	69	1	GCA_977946825	ERR14051044	ERR14041872
K44	SSI 81990	8,643,794	PacBio	28,320	11,510	4,938,150	50.73	56	2	GCA_977947275	ERR14051047	ERR14041874
K45	SSI 81991	10,978,198	PacBio	32,373	10,729	5,010,117	50.62	62	1	GCA_977947375	ERR14051121	ERR14041875
K46	SSI 81992	9,280,636	PacBio	32,459	10,445	4,805,835	50.8	63	0	GCA_977946895	ERR14051122	ERR14041877
K47	SSI 81993	10,409,706	PacBio	33,154	11,599	4,883,445	50.75	69	1	GCA_977947135	ERR14051123	ERR14041879
K48	SSI 81994	10,165,850	PacBio	23,045	10,255	4,960,040	50.74	41	1	GCA_977946915	ERR14051160	ERR14041881
K49	SSI 81995	9,169,518	PacBio	31,890	10,362	4,725,875	50.8	63	0	GCA_977946475	ERR14051161	ERR14041884
K50	SSI 81996	10,168,774	PacBio	31,925	10,689	5,081,262	50.66	59	1	GCA_977947205	ERR14051162	ERR14041886
K51	SSI 81997	8,834,586	PacBio	33,349	10,048	5,214,915	50.6	56	1	GCA_977947185	ERR14051202	ERR14041887
K52	SSI 81998	8,566,464	ONT	17,920	22,161	5,093,197	50.81	28	1	GCA_977947195	ERR14051208	ERR14041889
K53	SSI 81999	5,866,276	PacBio	35,373	9,496	5,110,598	50.55	60	0	GCA_977946495	ERR14051209	ERR14041894
K54	SSI 82000	11,910,714	PacBio	34,639	9,683	4,597,013	50.89	62	3	GCA_977946555	ERR14051210	ERR14041895
K55	SSI 82001	10,047,660	PacBio	37,216	9,746	4,661,405	51.02	70	1	GCA_977947805	ERR14051217	ERR14041897
K83	SSI 82003	9,893,698	PacBio	32,658	9,627	4,957,187	50.78	57	0	GCA_977946385	ERR14051218	ERR14041899
K84	SSI 82004	10,079,880	PacBio	35,163	10,084	4,949,990	50.74	62	3	GCA_977947815	ERR14051219	ERR14041901
K87	SSI 82005	8,400,154	PacBio	39,286	10,039	4,943,518	50.79	69	4	GCA_977946395	ERR14051220	ERR14041903
K92	SSI 82006	10,654,126	PacBio	33,033	10,439	5,156,189	50.58	58	2	GCA_977947695	ERR14051221	ERR14041906
K93	SSI 85376	7,285,978	PacBio	25,459	9,987	4,906,586	50.55	43	2	GCA_977946505	ERR14051222	ERR14041909
K94	SSI 85377	9,715,314	PacBio	35,202	10,259	4,886,020	50.71	66	0	GCA_977947735	ERR14051223	ERR14041911
K95	SSI 85378	13,358,338	PacBio	33,676	11,076	5,220,776	50.3	65	0	GCA_977947145	ERR14051224	ERR14041913
K96	SSI 85379	6,724,984	PacBio	28,338	11,097	5,063,914	50.63	54	1	GCA_977947645	ERR14051225	ERR14041914
K97	SSI 85380	6,641,816	ONT	30,822	26,522	5,298,499	50.74	30	5	GCA_977946295	ERR14051226	ERR14041915
K98	SSI 85381	13,814,536	PacBio	33,350	10,737	4,741,888	50.88	69	0	GCA_977947765	ERR14051229	ERR14041918
K100	SSI 82007	10,314,626	PacBio	33,594	9,707	5,098,252	50.62	57	1	GCA_977946755	ERR14051230	ERR14041920
K101	SSI 82008	10,089,768	PacBio	37,418	8,992	4,764,323	50.75	62	2	GCA_977946665	ERR14051231	ERR14041921
K102	SSI 82009	10,424,902	PacBio	37,305	9,394	4,726,166	50.73	64	3	GCA_977947395	ERR14051246	ERR14041925
K103	SSI 82010	10,278,752	PacBio	26,680	9,068	4,798,391	50.78	45	0	GCA_977946695	ERR14051248	ERR14041926

^*a*^ Strain identifiers are used by the Statens Serum Institut and the Culture Collection University of Gothenburg as part of their reference strain collections.

## Data Availability

The genomes and sequencing data reported in this study have been deposited at the European Nucleotide Archive under the project PRJEB83217 with accession numbers listed in Table 1. All scripts used to generate the reported assemblies are available on GitHub (https://github.com/carinemores/ecoliCPS).
